# Anatomical Reference of the Femur after Distal Resection Is Reliable for Rotational Alignment in Total Knee Arthroplasty

**DOI:** 10.3390/jpm14060663

**Published:** 2024-06-20

**Authors:** Seong Hwan Kim, Yong-Beom Park, Gil-Won Choi, Han-Jun Lee

**Affiliations:** 1 Department of Orthopaedic Surgery, Chung-Ang University Hospital, 102, Heukseok-ro, Dongjak-gu, Seoul 06973, Republic of Korea; ksh170177@nate.com (S.H.K.); gilwon07417@cauhs.or.kr (G.-W.C.); 2 Department of Orthopaedic Surgery, Chung-Ang University Gwangmyeong Hospital, Chung-Ang University College of Medicine, 110 Deokan-ro, Gwangmyeong-si 14353, Republic of Korea; whybe78@cau.ac.kr

**Keywords:** total knee arthroplasty, distal femur, rotational alignment, femoral rotation, anatomy

## Abstract

The rotational alignment of the femoral component in total knee arthroplasty (TKA) is considered an important factor, but it is still difficult to assess intraoperatively. This study was conducted to identify anatomical parameters for femoral rotational alignment. A total of 204 patients who underwent primary TKA between 2015 and 2019 were enrolled. The femoral lateral (FLAP) and femoral medial anteroposterior (FMAP) lengths were measured as the widest lengths in the anteroposterior (AP) axis after distal femoral resection. The difference between FLAP and FMAP was defined as dFAP. The concordance correlation coefficient (CCC) was assessed for agreement between the cTEA-PCA and the value of femoral rotation using the linear regression analysis equation. HKA, FLAP, FMAP, and dFAP were significantly associated with femoral rotational alignment. The prediction equation combining the novel intraoperative anatomical references showed improved association with rotational alignment. If dFAP was 6.0 mm, the femoral rotation angle was calculated as 4.9° using this univariate regression equation. The CCC was 0.483, indicating moderate agreement. The dFAP showed an association with distal femoral rotational alignment. A 6 mm dFAP could be a reference for around 5° of femoral rotation. The equation developed in this study may be a reliable tool for intraoperative distal femoral rotational alignment.

## 1. Introduction

The rotational alignment of the femoral component in total knee arthroplasty (TKA) is considered an important factor that can affect patellofemoral and tibiofemoral knee kinematics [1,2,3,4]. Excessive internal rotation of the femoral component in relation to the femoral posterior condylar axis can lead to knee pain and stiffness, which is caused by an increase in medial collateral ligament (MCL) tension and the gap between the femur and tibia on the lateral side [5,6]. To avoid these complications, surgeons often externally rotate the femoral component in an attempt to better balance the ligament and contact forces on the medial and lateral compartments and to restore the patellofemoral and tibiofemoral kinematics [7]. However, excessive external rotation could also lead to an imbalance of the soft tissue and mediolateral contact force, especially during flexion [8,9].

To avoid malrotation of the femoral component, several axes have been developed, including the Whiteside line [10], the surgical or clinical transepicondylar axis (TEA) [11], and a 3° external rotation of the posterior femoral condyles [12], to evaluate femoral component rotation during TKA. The Whiteside line could be easily used intraoperatively, but its reliability was reported to be low due to individual variations in the trochlea [10,13,14,15]. The 3° external rotation of the posterior femoral condyles could be used with higher reliability than that of the Whiteside line, but it may not reveal the differences in individual femoral rotation or the morphology of the distal femoral condyle [12,13,14,15]. The rotational alignment parallel to the TEA is generally accepted in the recently reported measured resection TKA technique [16,17], and is considered proper alignment for the balanced flexion gap in patellofemoral tracking [18]. However, identifying the anatomical landmarks of these reference axes intraoperatively is challenging and unreliable, regardless of conventional or navigated surgery [13,14,15,16], even though the reliability and reproducibility of the preoperative measurements of these angles have improved using computed tomography (CT) [19,20] or magnetic resonance imaging (MRI) [21,22]. Nevertheless, previous studies have focused on the diagnostic values or variations between the preoperative measured rotation angles on CT or MRI and the intraoperative measured angles determined using those reference axes [13,14,16,19]. The lateral and medial femoral condyles are asymmetrical; however, this is not reflected in the current TKA design during the determination of femoral component rotations and AP size [23,24,25,26]. There might be a reason for the differences between the reference angles on CT and the intraoperative measurements. Furthermore, the measured femoral rotational angle could be affected by the remnant cartilage of the lateral femoral condyle, which is known to cause an additional 1.5° of external rotation, although its clinical impact is limited [15,20]. To the best of our knowledge, the association between the distal femoral rotation angle and intraoperative anthropometric parameters after distal femoral resection has not been well investigated.

The purpose of this study was to (1) identify reproducible anatomical parameters of the distal femur after resection as a reference for rotational alignment of the distal femur during primary TKA and (2) investigate whether combining intraoperative anatomical parameters could predict femoral rotational alignment reliably. It was hypothesized that the intraoperative anatomical parameters after distal femoral resection would be associated with the rotational alignment of the distal femur measured by the clinical transepicondylar—posterior condyle axis angle (cTEA-PCA) on CT.

## 2. Methods

This study was conducted in accordance with the Declaration of Helsinki. This study was designed as a retrospective study, and the requirement for informed consent was waived by ethical committee of the Chung-Ang University Hospital Institutional Review Board. This study was approved by the Institutional Review Board of Chung-Ang University Hospital IRB (protocol code IRB no. 2003-001-19303) and it was confirmed that all research was performed in accordance with the relevant guidelines. This is a retrospective cohort study of 386 patients who underwent primary TKA between 2015 and 2019. Patients with osteoarthritis (OA) with varus knee were included in this study, whereas those with valgus knees, congenital deformities, such as lateral femoral condylar hypoplasia, a history of previous surgery, bony defects, severe wear on the medial femoral condyle (ICRS grade 4), or those in whom the femoral epicondyles could not be seen accurately on CT owing to spurs or deformities were excluded from the study. After applying the inclusion/exclusion criteria, 204 patients were included in this study.

### 2.1. Radiographic Measurements

**Figure 1:**
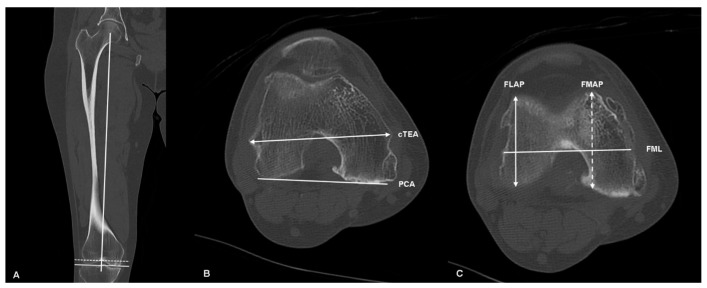
Radiographic measurements on computed tomography. (**A**) Determination of the distal femoral resection 9 mm proximal to the lowest point of the femoral condyle perpendicular to the mechanical axis of the femur. (**B**) The clinical transepicondylar axis and posterior condyle axis angles were drawn to assess distal femoral rotational alignment. (**C**) The anteroposterior length of the lateral femoral condyle (FLAP), the anteroposterior length of the medial femoral condyle (FMAP), and the mediolateral femoral length (FML) were measured.

Preoperative radiographic measurements were obtained for the rotational alignment of the distal femur using CT (1.2-mm slice thickness, GE Healthcare, Waukesha, WI, USA). The distal femoral scan was performed for each knee in the 30° flexion position, with the scan direction set perpendicular to the anatomical axis of the femur. All radiographic parameters on CT were measured using the image taken 9 mm proximal from the lowest point of the femoral condyle perpendicular to the mechanical axis of the femur, as was performed on the cutting surface of the distal femur intraoperatively (Figure 1A). The cTEA was defined as a line connecting the most prominent points on the medial and lateral epicondyles of the femur (Figure 1B). The PCA was defined as a line connecting the most prominent points on the medial and lateral femoral posterior condyles (Figure 1B). The angle of the cTEA relative to the PCA (cTEA-PCA), measured on the CT, was defined as femoral rotational alignment [15]. The femoral lateral anteroposterior (FLAP) and femoral medial anteroposterior (FMAP) lengths were defined as the widest aspects of the lateral and medial femoral condyles in the AP axis at the level of the distal femoral resection of 9 mm, based on the mechanical axis of the femur (Figure 1C) [27,28]. The difference between FLAP and FMAP (dFAP) was defined as FMAP minus FLAP. The mediolateral femoral length (FML) was measured at the widest aspect along the cTEA (Figure 1C). The mechanical hip–knee–ankle axis angle (HKA), the mechanical medial proximal tibial angle (MPTA), and the mechanical lateral distal femoral angle (LDFA) were measured on the radiograph [25,29]. The varus alignment was set as positive for HKA.

### 2.2. Measurement of the Intraoperative Anatomical Parameters

**Figure 2:**
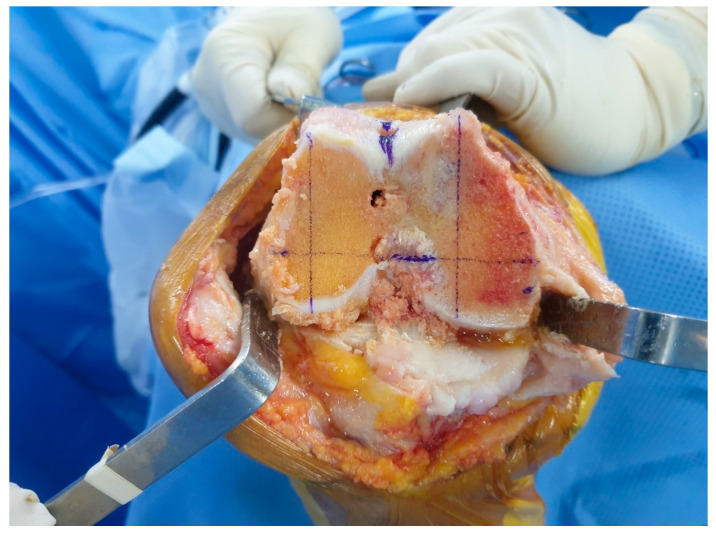
Intraoperative measurements of the anteroposterior length of the lateral femoral condyle (FLAP) and the anteroposterior length of the medial femoral condyle (FMAP).

All primary TKAs were performed using the measured resection technique by one senior surgeon. Distal femoral resection was performed with a distal resection thickness of 9 mm, based on the mechanical axis of the femur. After distal femoral resection, FLAP, FMAP, and FML were outlined on the surface of the distal femoral resection and measured using a Vernier caliper intraoperatively (Figure 2).

### 2.3. Statistical Analysis

All statistical analyses were performed using SPSS for Windows, version 19.0 (SPSS, Chicago, IL, USA) and G-power (ver. 3.1.5, Düsseldorf University). The predictive equation for the outcome variable (cTEA-PCA) was developed using linear regression analysis. We accepted two-sided α-errors of 5% and β-errors of 20% to detect any significant difference. The post-hoc power analysis for the primary outcome involved determining the effect size using Cohen’s f2 equation, using the adjusted R^2^ value from the linear regression analysis. The observed statistical power of the linear regression analysis for predicting cTEA-PCA was calculated as 1.0, with an effect size of 0.32275132.

Independent and paired *t*-tests were used to compare the mean data. The continuous values were analyzed for normality using the Shapiro–Wilk test. Univariate and multivariate linear regression analyses with stepwise methods were used to find the equation of the predictor variables (demographic and radiographic measurements) in predicting the outcome variable (cTEA-PCA). There may have been around 1.5° of additional external rotation due to the remnant cartilage of the lateral femoral condyle. We also incorporated this value into the final equation [15,20]. The concordance correlation coefficient (CCC) was assessed for agreement between the cTEA-PCA and the estimated value of femoral rotation using the linear regression analysis equation [30].

To validate the measurements of FLAP and FMAP between the radiologic (CT) and the intraoperative bone surface, the Bland–Altman plot and its 95% limits of agreement (95% LOA) were evaluated [30,31]. The intra- and inter-observer reliability of the two orthopedic surgeons was retested 2 weeks after the first assessment, and the average values were used. The reliabilities of the radiological measurements were assessed by calculating the intraclass correlation coefficients (ICCs).

## 3. Results

### 3.1. Subject Characteristics

The overall demographics and radiologic parameters are summarized in Table 1.

### 3.2. Associated Anatomical Parameters with Rotational Alignment of the Distal Femur and Its Prediction Models

**Figure 3:**
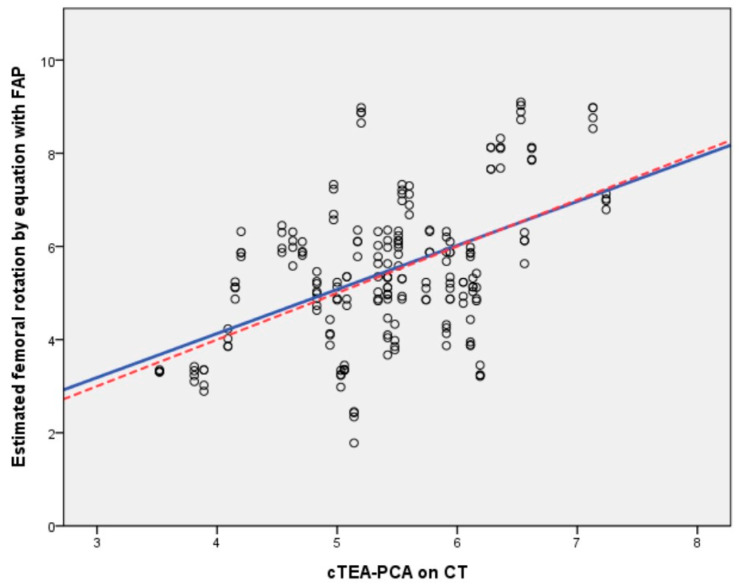
Scatter plot for femoral rotational alignment on computed tomography and estimated femoral rotation using the equation with dFAP (dFAP, difference between the anteroposterior length of the lateral femoral condyle (FLAP) and anteroposterior length of the medial femoral condyle (FMAP)).

Univariate and multivariate linear regression analyses were performed to find the radiologic parameters associated with femoral rotational alignment (cTEA-PCA) (Table 2). In the univariate linear regression analysis, HKA, FLAP, FMAP, and dFAP were significantly associated with femoral rotational alignment. FMAP and dFAP showed higher adjusted R^2^ values than the other parameters. Due to interactions among FLAP, FMAP, and dFAP, these parameters were included separately in the multivariate linear regression analysis. In the multivariate linear regression analysis, dFAP and HKA were significantly associated with femoral rotation alignment, and FMAP, FLAP, FML, and HKA were found to be significant associated factors. Regarding clinical relevance, the model using dFAP was easier to use intraoperatively than the models using FLAP and FMAP; hence, the prediction model using dFAP was chosen, considering the 1.5° of excessive external rotation due to lateral femoral condylar cartilage. The prediction equation was developed using dFAP in the multivariate linear regression analysis as follows: femoral rotational alignment = 4.8 + [0.298×dFAP] − [0.043×HKA] − 1.5°. In the univariate linear regression analysis using dFAP, the prediction equation was as follows: femoral rotational alignment angle = 4.624 + [0.301×dFAP] − 1.5°. If dFAP was 6.0 mm, the femoral rotation angle was calculated as 4.9° using this univariate regression equation. The CCC was also calculated to evaluate the agreement between the cTEA-PCA on CT and the estimated femoral rotation using the univariate and multivariate regression equations with dFAP. The CCC was 0.483 (95% confidence interval (CI): 0.389–0.569) for the univariate regression analysis and 0.525 (95%CI: 0.442–0.604) for the multivariate regression analysis, all showing moderate agreement (Figure 3).

### 3.3. Validation between Radiologic Parameters and Intraoperative Measurements

**Figure 4:**
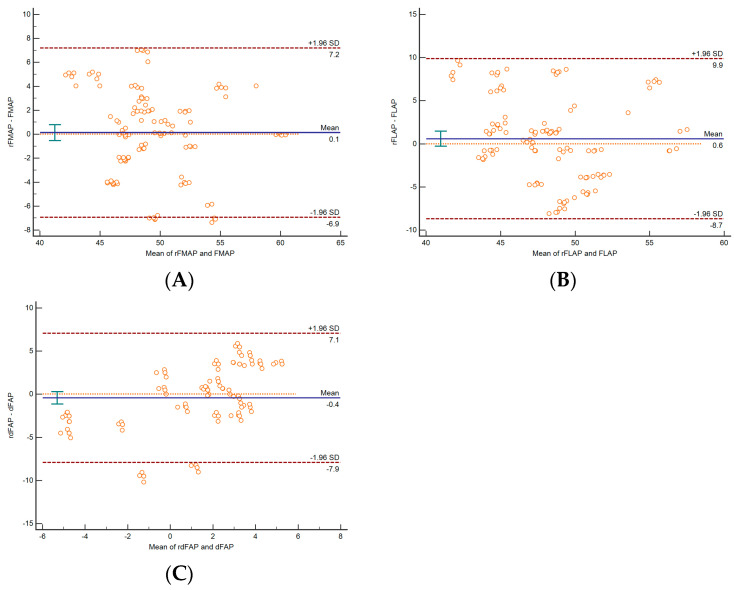
The Bland–Altman plots are shown to validate the values between the measurements obtained using computed tomography and those obtained intraoperatively. (**A**) Plot for the anteroposterior length of the medial femoral condyle. [(rFMAP: intraoperative FMAP) - (FMAP)]. (**B**) Plot for the anteroposterior length of the lateral femoral condyle. [(rFLAP: intraoperative FLAP) - (FLAP)]. (**C**) Plot for the difference between FLAP and FMAP. [(rdFAP: intraoperative dFAP) - (dFAP)].

The mean values of FMAP, FLAP, and dFAP were not significantly different between the radiologic and intraoperative measurements, although FLAP was larger intraoperatively (Table 3). The mean values of FMAP, FLAP, and dFAP demonstrated high correlation between the radiologic and intraoperative measurements (Table 3). The Bland–Altman plots revealed acceptable mean differences, with a difference of 0.6 mm in FLAP due to cartilage thickness (Table 4, Figure 4A–C).

## 4. Discussion

The most important finding from this study is that the anatomical references of FMAP and dFAP after distal femoral cutting were associated with rotational alignment of the distal femur. Additionally, the increased dFAP, elongated FMAP, decreased FLAP and HKA, and narrow FML were also correlated with increased femoral external rotation alignment. Moreover, around 6 mm of intraoperative dFAP could be used as the clinical cut-off value to assess external femoral rotation around 5°.

Rotational alignment of the femoral component in TKA is one of the important factors affecting knee kinematics and clinical outcomes [1,2,3,4]. To obtain proper femoral component rotation, the intraoperative assessment of femoral rotation is essential, and the preoperative measurement of femoral rotation should also be obtained before TKA, especially when using the measured resection technique [1,2,3,4]. Thus, several anatomical references of the distal femur have been considered for femoral component rotation during TKA. However, identification of the intraoperative landmarks of these reference points has been challenging [13,14,15,16]. Although many intraoperative methods have been used to assess femoral rotation [16,18,19], the reproducibility and reliability of these methods have been reported to be low for their use as standard intraoperative measurements [11,13,14,15,32,33,34]. Thienpont et al. [32] found a high rate (41%) of misalignment when surgeons used a fixed angle of 4° between the posterior condylar axis and TEA during measured resection TKA. Furthermore, the remnant cartilage of the lateral femoral condyle also complicates the measurement of a reliable intraoperative femoral rotation angle compared to the femoral rotation angle on CT [15,20]. Although the clinical impact of the additional external femoral rotation angle by the remnant cartilage of the lateral femoral condyle is known to be limited [15], it could still induce differences in the measured femoral rotation angles between intraoperative and preoperative CT, which can also be unreliable [15,20]. Thus, to determine accurate femoral rotation intraoperatively, it would be necessary to utilize a combination of other factors and methods, as well as precise preoperative and intraoperative measurements of femoral rotation, including consideration of the remnant cartilage of the femoral condyle [15,16,20].

In this study, FMAP and dFAP were associated with rotational alignment of the distal femur. Distal femoral morphology varies according to sex, ethnicity, or individual factors [24,25,35,36]. In previous studies, femoral posterior condyles have been reported to be asymmetrical in width, with the lateral side being smaller than the medial [24], narrower among women compared to men [24,35,36], and longer anteroposteriorly among the Black population compared to the Asian population [25,28]. Thus, we assumed that femoral rotation might be correlated with these morphological features, especially the anteroposterior length of the medial and lateral femoral condyle or the mediolateral length. In brief, according to the results of this study, FMAP or dFAP may be a useful supplementary anatomical reference to determine intraoperative femoral component rotation during primary TKA. Furthermore, this novel anatomical reference could give more information regarding rotational alignment after distal femur resection, which would be difficult to determine after resection.

The prediction equation developed in this study, combining novel intraoperative anatomical references, showed improved association with femoral rotational alignment. Therefore, this novel method, which has good reliability and validity, may be used as a supplemental tool to assess intraoperative femoral rotation, which is otherwise difficult to assess, especially after distal femoral resection. Increased dFAP and a decreased HKA angle were associated with the femoral rotation angle in the linear regression analysis, and the narrow femoral condyle with elongated FMAP was also found to be a predictive factor. Moreover, a dFAP of approximately 6 mm could be used as a cut-off to assess femoral external rotation around 5°. Thus, the intraoperative femoral rotation angle could be confirmed and compared using preoperative femoral rotation planning on CT with this novel method before 4-in-1 block resection, which is difficult to assess intraoperatively. Finally, the reliability and validity evaluated by ICC and the mean differences in the Bland–Altman plot were found to be highly acceptable between the radiologic and intraoperative measurements. Thus, this novel method can provide reliable and reproducible intraoperative references for distal femoral rotational alignment during primary TKA. According to the results of this study, femoral rotational alignment could differ with the size of the distal femur, suggesting its potential utility as a supplementary reference during non-conventional techniques, such as robot-assisted TKA, where determining femoral component rotation with traditional references may be challenging [37]. 

This study has several limitations. First, patients with valgus knee or lateral femoral condyle hypoplasia were excluded from this study; hence, the results may vary in patients with valgus knees. Furthermore, only patients without any deformities of the lateral femoral condyle were included, and the femoral rotation angle was affected by FMAP but not FLAP. Thus, dFAP would be more appropriate to use intraoperatively under general conditions. However, according to the results of this study, it could be assumed that patients with a narrow distal femoral condyle might have higher femoral external rotation (e.g., females or Black people), and care should be taken during TKA for these patients [23,25,36]. Furthermore, there may be associations between CPAK and distal femoral morphologies, which could affect femoral rotational alignment [38,39,40]. Second, the results of this study were obtained using an East Asian population and there may be differences among other ethnicities [25,28]. However, dFAP could be used if the analysis was performed for each ethnic population, even though the equation would differ. Third, considerable individual variations, according to distal femoral anatomy or cartilage thickness, were observed, as well as differences owing to the use of different reference axes, such as the surgical or clinical TEA [6,8,9,21,32,33,34]. Further, there may be errors according to the cartilage status of the posterior femoral condyle [6,15,21]. In this study, the intraoperative FLAP was larger than that following radiologic measurement, although this was not significant (Table 3 and Table 4). The values obtained using CT could not reveal the exact intraoperative cartilage thickness. However, we also considered additional femoral external rotation due to the remnant cartilage of the lateral femoral condyle, which may be addressed in the equation of this study.

## 5. Conclusions

In conclusion, there is an increased possibility of excessive external femoral rotational alignment in patients with increased dFAP and decreased HKA. Patients with a dFAP of approximately 6 mm may have femoral external rotation around 5°, and the equation developed in this study could be used as a reliable supplementary reference to assess intraoperative distal femoral rotational alignment during primary TKA, which is difficult to evaluate intraoperatively after distal femoral resection.

## Figures and Tables

**Table 1:** Patient Demographics (Mean ± Standard Deviation).

	Overall Results
Patients (number)	204
Sex (Male/Female)	29:175
Age (y)	69.8 ± 7.7
BMI	26.4 ± 3.5
Degree of osteoarthritis (Kellgren and Lawrence Score)	3.5 ± 1.0
cTEA-PCA	5.4° ± 2.1
Mechanical HKA angle	varus 9.2° ± 4.7
MPTA	84.1° ± 5.5
LDFA	88.6° ± 2.5
FLAP	52.4 mm ± 3.9
FMAP	55.2 mm ± 3.8
dFAP	2.8 mm ± 3.6
FML	68.2 mm ± 4.7

BMI: body mass index, cTEA: clinical transepiconylar axis, PCA: posterior condylar axis, HKA: hip–knee–ankle axis, FLAP: femoral lateral anteroposterior length, FMAP: femoral medial anteroposterior length, dFAP: difference between FLAP and FMAP, FML: mediolateral femoral length, MPTA: mechanical medial proximal tibial angle, LDFA: mechanical lateral distal femoral angle.

**Table 2:** The Univariate and Multivariate Linear Regression Analysis for Femoral Rotational Alignment.

	cTEA-PCA		
	ß ± SE	*p*-Value	Adjusted R^2^
Univariate linear regression analysis			
1. HKA	−0.056 ± 0.033	0.091	0.028
2. MPTA	−0.008 ± 0.024	0.741	0.001
3. LDFA	−0.085 ± 0.049	0.084	0.015
4. FLAP	0.134 ± 0.037	<0.001	0.06
5. FMAP	0.264 ± 0.025	<0.001	0.346
6. dFAP	0.301 ± 0.035	<0.001	0.242
7. FML	0.012 ± 0.024	0.637	0.001
Multivariate linear regression analysis including dFAP			
Intercept	4.8 ± 0.228		0.256
dFAP	0.298 ± 0.035	<0.001	
HKA	−0.043 ± 0.021	0.043	
Multivariate linear regression analysis including FMAP and FLAP			
Intercept	−3.872		0.409
FMAP	0.357 ± 0.033	<0.001	
FLAP	−0.124 ± 0.039	0.002	
FML	−0.05 ± 0.02	0.016	
HKA	−0.046 ± 0.019	0.017	

cTEA: clinical transepiconylar axis, PCA: posterior condylar axis, HKA: hip–knee–ankle axis, FLAP: femoral lateral anteroposterior length, FMAP: femoral medial anteroposterior length, dFAP: difference between FLAP and FMAP, FML: mediolateral femoral length, MPTA: mechanical medial proximal tibial angle, LDFA: mechanical lateral distal femoral angle.

**Table 3:** Analysis of the Correlation between the Radiologic and Intraoperative Measurements in all Patients.

	Mean Value ± Standard Deviation		*p*-Value	ICC	95% CI	*p*-Value
	Radiologic	Intraoperative				
FMAP	55.2 mm ± 3.8	55.4 mm ± 4.6	0.831	0.811	0.684–0.935	0.001
FLAP	52.4 mm ± 4.1	53.0 mm ± 4.9	0.35	0.803	0.665–0.928	0.001
dFAP	2.8 mm ± 3.6	2.4 mm ± 4.2	0.302	0.779	0.623–0.872	0.008

FLAP: femoral lateral anteroposterior length, FMAP: femoral medial anteroposterior length, dFAP: difference between FLAP and FMAP.

**Table 4:** Mean Difference and 95% Limits of Agreement (95% LOA) between Radiologic and Intraoperative Measurements in the Bland–Altman Analysis for Validity.

	Differences between Intraoperative and Radiographic Measurements	
	Mean Difference	95% LOA
FMAP	0.1 mm	−6.9 mm ~ 7.2 mm
FLAP	0.6 mm	−8.7 mm ~ 9.9 mm
dFAP	−0.4 mm	−7.9 mm ~ 7.1 mm

FLAP: femoral lateral anteroposterior length, FMAP: femoral medial anteroposterior length, dFAP: difference between FLAP and FMAP.

## Data Availability

All data are available from the corresponding author upon request.
